# External Apical Root Resorption in Vital and Endodontically Treated Teeth Following Fixed Orthodontic Treatment: A Retrospective Longitudinal Panoramic Study

**DOI:** 10.3390/jcm15051963

**Published:** 2026-03-04

**Authors:** Nuri Can Tanrısever, Mehmet Okan Akçam

**Affiliations:** Department of Orthodontics, School of Dentistry, University of Ankara, 06560 Ankara, Turkey

**Keywords:** external apical root resorption, orthodontic treatment, endodontically treated teeth, panoramic radiography

## Abstract

**Objective**: External apical root resorption is a frequent complication of orthodontic treatment, and the response of endodontically treated teeth remains controversial. This study aimed to compare external apical root resorption (EARR) in endodontically treated teeth and vital teeth following fixed orthodontic treatment in patients with Angle Class I, II, and III malocclusions using digital panoramic radiography. **Methods**: This retrospective longitudinal study included 60 patients (mean age: 16.3 ± 2.4 years) who underwent non-extraction fixed orthodontic treatment. A paired contralateral within-subject design was used, whereby each patient contributed one endodontically treated tooth and its symmetrical untreated vital counterpart. Root length was measured on calibrated panoramic radiographs obtained before (T0) and after treatment (T1). Differences were analyzed using the Wilcoxon signed-rank and Kruskal–Wallis tests (*p* < 0.05). **Results**: Both endodontically treated and vital teeth exhibited statistically significant reductions in root length between T0 and T1 (mean reduction: 1.02 ± 1.36 mm and 1.11 ± 1.79 mm, respectively; *p* < 0.001). No significant difference was observed between the two tooth types regarding the magnitude of resorption. Similarly, no significant differences were detected among Angle Class I, II, and III malocclusion groups. The observed mean reduction of approximately 1 mm suggests limited apical shortening within the range generally considered clinically moderate. **Conclusions**: Endodontically treated teeth exhibited a degree of EARR comparable to that of vital teeth following fixed orthodontic treatment, suggesting that properly treated endodontic teeth do not pose an increased risk of clinically relevant apical root resorption during orthodontic therapy.

## 1. Introduction

External apical root resorption (EARR) is one of the most common undesirable side effects associated with orthodontic tooth movement and represents a complex, irreversible pathological process affecting the external cementum and root dentin [1,2]. The primary etiological factor of EARR is the application of orthodontic forces that exceed the physiological tolerance of the periodontal ligament, triggering a cascade of biological responses [3]. Excessive or sustained pressure leads to localized hyalinization of the periodontal ligament, followed by the recruitment of clastic cells, ultimately resulting in resorptive defects on the root surface [4,5].

EARR is considered a multifactorial phenomenon influenced by a combination of genetic susceptibility, individual biological variability, and mechanical factors [6,7]. Brezniak et al. reported that the development and severity of EARR during orthodontic treatment are affected by systemic and genetic factors, root morphology, alveolar bone characteristics, as well as the type, direction, magnitude, and duration of applied orthodontic forces [8].

Histological studies have demonstrated that some degree of EARR occurs in up to 90% of teeth subjected to orthodontic forces [9,10]. However, clinically significant resorption, defined as the loss of approximately one-third of the root length, has been reported in only 1–5% of affected teeth [11].

Clinically, EARR is usually asymptomatic and is detected through routine radiographic examinations, including panoramic, periapical, and lateral cephalometric radiographs obtained during orthodontic treatment [12]. Although cone-beam computed tomography (CBCT) allows three-dimensional and more detailed visualization of resorptive defects, conventional radiographic methods remain widely used in clinical orthodontic practice for longitudinal evaluation due to lower radiation exposure and broader availability [13].

In clinical orthodontic practice, smaller degrees of apical shortening (e.g., approximately 1–2 mm) are generally considered mild to moderate and may not necessarily compromise tooth prognosis. Therefore, in the present study, the evaluation focused on quantitative linear changes in root length rather than exclusively on severe resorption thresholds, in order to assess relative differences between endodontically treated and vital teeth.

With increasing public awareness and advancements in orthodontic techniques, the number of patients undergoing orthodontic treatment in adolescence and adulthood has steadily increased, leading to a higher prevalence of endodontically treated teeth encountered during treatment. The biological response of endodontically treated teeth to orthodontic forces has long been debated. Previous studies evaluating histological reactions and EARR have reported comparable responses between endodontically treated (ETT) and untreated vital teeth (VT) with no history of endodontic therapy [14]. Nevertheless, the existing literature remains inconclusive: some studies have reported greater EARR in endodontically treated teeth [15,16], whereas others have found no significant differences [17], or even less resorption compared with vital teeth [18,19].

Despite the growing body of literature, previous studies have reported inconsistent findings and have often evaluated endodontically treated and vital teeth without strict contralateral within-subject comparison or stratification according to Angle malocclusion classification. In particular, evidence assessing external apical root resorption across Class I, II, and III malocclusion groups under routine clinical conditions remains limited. Addressing these gaps may provide a more controlled and clinically applicable understanding of whether endodontic status influences the extent of orthodontically induced root resorption.

Therefore, the aim of the present study was to evaluate and compare external apical root resorption in vital and endodontically treated teeth using digital panoramic radiography following fixed orthodontic treatment in patients with Angle Class I, II, and III malocclusions.

The null hypothesis of this study was that there would be no statistically significant difference in the magnitude of external apical root resorption between endodontically treated teeth and vital teeth following fixed orthodontic treatment.

## 2. Materials and Methods

### 2.1. Study Design and Ethical Approval

This retrospective longitudinal study was conducted using archived panoramic radiographs obtained before (T0) and after (T1) fixed orthodontic treatment. The radiographs were retrieved from routine patient records archived between October 2023 and December 2024. Ethical approval for the retrospective analysis of anonymized radiographic data was granted by the Institutional Ethics Committee (Meeting No: 6; Approval Date: 10 February 2025) prior to data extraction and statistical analysis.

Due to the retrospective nature of the study and the use of fully anonymized data, the requirement for informed consent was waived by the Ethics Committee. All procedures were carried out in accordance with the Declaration of Helsinki.

This study was designed and reported in accordance with the STROBE guidelines for observational cohort studies. A completed STROBE checklist is provided as Supplementary Material.

### 2.2. Study Sample

The study sample consisted of panoramic radiographs of 60 patients (mean age: 16.3 ± 2.4 years; 33 males, 27 females) with Angle Class I, II, and III malocclusions who underwent non-extraction fixed orthodontic treatment. All radiographs were obtained under standardized conditions using the same panoramic radiographic device (Carestream Dental 8100, Carestream Health Inc., Rochester, NY, USA), with the head positioned in a neutral posture and acquired by the same radiology technician.

Each patient contributed only one pair of symmetrical contralateral teeth, consisting of one endodontically treated tooth and its untreated vital counterpart, thereby ensuring statistical independence of observations. When more than one eligible contralateral pair was present in the same patient, a single pair was selected based on fulfillment of inclusion criteria and radiographic clarity.

Only contralateral teeth of the same tooth type (e.g., molar–molar, premolar–premolar, canine–canine, incisor–incisor) were included to ensure anatomically and biomechanically comparable measurements.

The included tooth pairs comprised incisors, canines, premolars, and molars located in both the maxilla and mandible.

The distribution of tooth types included in the study comprised 23 molars, 17 premolars, 11 canines, and 9 incisors.

A total of 60 patient records fulfilling the predefined inclusion criteria were identified from the archived database and included in the final analysis. No additional exclusions were required after radiographic quality assessment.

### 2.3. Inclusion and Exclusion Criteria

Patients were included if they met the following criteria:

- No history of previous orthodontic treatment,
- Completed root development prior to orthodontic treatment (excluding third molars),
- Presence of symmetrical contralateral teeth, one endodontically treated and one vital,
- Absence of radiographic evidence of active periapical inflammation, periapical radiolucency, or other signs of endodontic pathology at T0.

Additionally, endodontically treated teeth were required to have completed root canal therapy at least six months prior to the initiation of orthodontic treatment. Treatment success was assessed radiographically and defined as the presence of a homogenous root canal filling extending to within 0–2 mm of the radiographic apex, without periapical radiolucency, widening of the periodontal ligament space, or visible voids within the filling material.

Teeth with compromised incisal or occlusal edge integrity, poor radiographic image quality, or unclear cemento-enamel junction (CEJ) delineation were excluded from the study.

### 2.4. Orthodontic Treatment Protocol

All patients were treated using a standardized fixed orthodontic protocol without extractions, employing the 0.018-inch Roth prescription appliance system. Orthodontic treatment duration averaged 20.3 ± 2.1 months across all malocclusion groups.

Due to the retrospective archival design of the study, detailed records regarding the magnitude, direction, duration, and specific mechanics of applied orthodontic forces (e.g., torque control, intrusion mechanics, intermaxillary elastics, or skeletal anchorage systems) were not consistently available. Therefore, the analysis was limited to overall treatment duration and radiographic comparisons between T0 and T1.

### 2.5. Radiographic Measurements

**Figure 1:**
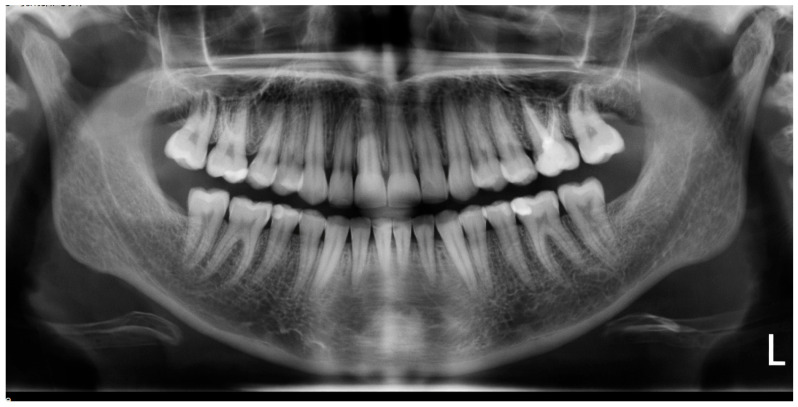
Representative digital panoramic radiograph obtained under standardized positioning conditions (Carestream Dental 8100). A root canal treated tooth is visible.

Panoramic radiographs (Figure 1) were obtained using the same panoramic device (Carestream Dental 8100) under standardized positioning conditions to ensure measurement consistency. All images were acquired according to the manufacturer’s protocol, with the Frankfort horizontal plane parallel to the floor.

Linear tooth length measurements were performed on digital panoramic radiographs according to the method described by Spurrier et al. [20], using reproducible anatomical landmarks identified at both T0 (pre-treatment) and T1 (post-treatment).

**Figure 2:**
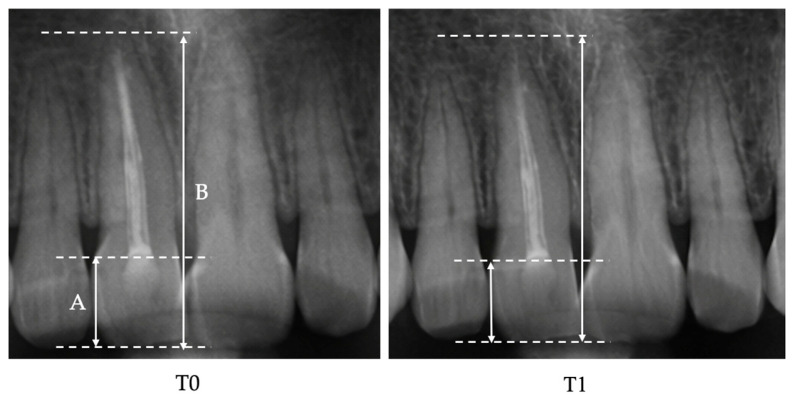
Measurement method for root length assessment in an endodontically treated tooth. A. Crown length measured as the longest distance between the incisal edge and the cemento-enamel junction (CEJ). B. Total tooth length measured as the longest inciso-apical distance from the incisal edge to the radiographic root apex.

The following linear measurements were recorded (Figure 2):

- Crown length: the longest distance between the incisal edge and the cemento-enamel junction (CEJ).
- Total tooth length: the longest inciso-apical distance from the incisal edge to the radiographic root apex.

For multi-rooted teeth, measurements were based on the longest root to ensure consistency in longitudinal evaluation.

The figure represents a magnified cropped section of the original panoramic radiograph. The image was enlarged for illustrative clarity only; all quantitative measurements were performed on the original-resolution panoramic images prior to magnification.

Measurements were performed digitally in AutoCAD 2025 (Autodesk Inc., San Francisco, CA, USA) by a single calibrated investigator (N.C.T.). Prior to export, images were calibrated within the native Carestream Dental software using the embedded calibration tool. Identical scaling parameters were maintained for both T0 and T1, and the same calibration reference was applied during AutoCAD analysis.

To assess potential magnification differences between time points, crown length measurements were evaluated as an internal control parameter. Because true crown length is not expected to change during orthodontic treatment in the absence of incisal wear or restorative procedures, stability of crown measurements supports consistency of image scaling. No significant difference was observed between T0 and T1 crown measurements (*p* > 0.05), indicating that magnification error was systematic rather than differential. Therefore, no additional magnification correction formula was applied.

To minimize the potential influence of tooth angulation changes between T0 and T1, all linear measurements were performed along the long axis of the tooth, defined by a line connecting the incisal edge and the radiographic root apex. The CEJ midpoint (between mesial and distal enamel margins) was used to standardize landmark identification.

Apical root resorption was calculated as the absolute linear difference (in millimeters, mm) between T0 and T1 total tooth length measurements:

Δ Root Resorption (mm) = (Inciso-apical length at T0) − (Inciso-apical length at T1)

A positive value indicates reduction in root length consistent with apical root resorption.

Although panoramic radiographs inherently involve geometric distortion, intra-individual comparisons under identical acquisition and calibration parameters minimize differential magnification bias.

The presence of root canal filling material did not interfere with measurements, as only external anatomical landmarks were used.

### 2.6. Statistical Analysis

Statistical analysis was performed using SPSS software (version 26.0; IBM Corp., Armonk, NY, USA).

An a priori power analysis was performed using G*Power software (version 3.1, Heinrich Heine University, Düsseldorf, Germany). The primary comparison involved paired measurements between T0 and T1 within the same subjects. Based on an expected large effect size (d = 0.60), an alpha level of 0.05, and a statistical power of 95% (1–β = 0.95), the minimum required sample size was calculated as 32 subjects. The final sample of 60 patients exceeded this requirement.

In the absence of pilot data or consistent effect estimates from previous contralateral within-subject studies, the expected effect size (d = 0.60) was selected a priori to represent a clinically meaningful between-group difference for sample size planning, following conventional benchmarks implemented in G*Power. The study was therefore powered to detect moderate-to-large differences considered clinically relevant.

Intra-observer reliability was assessed by re-measuring 20 randomly selected radiographs after a two-week interval, and intraclass correlation coefficients (ICC) were calculated.

A two-way mixed-effects model with absolute agreement definition (single measures) was used for ICC calculation. Ninety-five percent confidence intervals (95% CI) were also computed.

Data distribution was evaluated using skewness and kurtosis values (±2). In addition, normality was assessed using the Shapiro–Wilk test. Since the data did not meet parametric assumptions, non-parametric statistical tests were applied accordingly.

Paired comparisons between T0 and T1 measurements were performed using the Wilcoxon signed-rank test, both for the overall sample and separately within each Angle malocclusion classification. Comparisons among Angle Class I, II, and III malocclusion groups were conducted using the Kruskal–Wallis test. Differences between T0 and T1 (change scores), as well as intergroup comparisons related to these changes, were also analyzed using the Kruskal–Wallis test.

Additional subgroup analyses were performed to evaluate potential differences according to gender, tooth type, and jaw location. Gender- and jaw-based comparisons (maxilla vs. mandible) were conducted using the Mann–Whitney U test, while differences among tooth categories (molars, premolars, canines, and incisors) were analyzed using the Kruskal–Wallis test.

For the primary between-group comparison (ETT vs. VT change scores), effect size was calculated using Cohen’s d based on pooled standard deviation. Although non-parametric tests were used for hypothesis testing due to distributional characteristics of the data, Cohen’s d was reported as a standardized measure of effect magnitude to quantify the mean difference between groups and to facilitate clinical interpretation. The calculation of Cohen’s d was independent of the inferential test selection and was used descriptively to express the magnitude of the observed difference.

Additionally, 95% confidence intervals (CI) were computed for mean change values and for the between-group mean difference.

Each patient contributed only one contralateral tooth pair; therefore, the patient was considered the unit of analysis. Because no patient contributed multiple independent pairs, multilevel or clustering adjustment was not required. Statistical significance was set at *p* < 0.05.

## 3. Results

Intra-observer reliability was excellent, with intraclass correlation coefficient (ICC) values greater than 0.90.

The distribution of tooth types and jaw location is presented in Table 1. Of the 120 teeth evaluated (60 endodontically treated and 60 contralateral vital teeth), 58 teeth were located in the maxilla and 62 in the mandible. Molars constituted the largest subgroup (n = 46), followed by premolars (n = 34), canines (n = 22), and incisors (n = 18).

Because each patient contributed only one contralateral pair of the same tooth type located in the same jaw, anatomical and biomechanical comparability between endodontically treated and vital teeth was maintained.

In intra-group comparisons, both endodontically treated teeth (ETT) and vital teeth (VT) exhibited statistically significant reductions in root length between T0 and T1 (*p* < 0.001 for both).

These findings confirm that external apical root resorption occurred following fixed orthodontic treatment irrespective of endodontic status.

Root length measurements were significantly greater at T0 than at T1 in both groups (Table 2).

When the magnitude of change between T0 and T1 was compared between endodontically treated and vital teeth, no statistically significant difference was observed (Table 3), indicating a similar extent of apical root resorption in both groups. The between-group mean difference in root length reduction (ETT − VT) was −0.09 mm (95% CI: −0.66 to 0.48), with a negligible effect size (Cohen’s d = 0.06).

The 95% confidence interval included zero and was relatively narrow, further supporting the absence of a statistically significant and clinically meaningful difference between the two groups.

In addition to mean ± SD values, median and interquartile range (IQR) values were calculated for the primary change scores to appropriately summarize the non-normally distributed data. The median root length reduction was 0.75 mm (IQR: 0.18–1.45) in the endodontically treated teeth and 0.50 mm (IQR: 0.10–1.48) in the vital teeth. The overlapping interquartile ranges further support the similarity between groups.

Within the overall sample, Spearman correlation analysis demonstrated no statistically significant association between individual treatment duration and root length reduction in endodontically treated teeth (r = −0.022, *p* = 0.868) or in vital teeth (r = −0.140, *p* = 0.287).

When subgroup analyses were performed according to gender, no statistically significant differences in apical root resorption were observed between male (n = 33) and female (n = 27) patients in either the endodontically treated or vital tooth groups (Mann–Whitney U test, *p* > 0.05).

Similarly, no statistically significant differences were detected in the magnitude of root length reduction according to jaw location (maxilla vs. mandible) in either group (Mann–Whitney U test, *p* > 0.05). Comparisons across tooth types (molars, premolars, canines, and incisors) also demonstrated no statistically significant differences in resorption magnitude (Kruskal–Wallis test, *p* > 0.05).

These findings suggest that the observed root length reduction was consistent across sex, arch location, and tooth type categories within the limitations of the present sample.

Within each Angle malocclusion group, paired comparisons between T0 and T1 demonstrated statistically significant reductions in root length in endodontically treated teeth (Class I: *p* = 0.011; Class II: *p* = 0.002; Class III: *p* < 0.001) as well as in vital teeth (Class I: *p* = 0.005; Class II: *p* = 0.022; Class III: *p* = 0.001) (Wilcoxon signed-rank test). In all malocclusion classes, root length measurements were significantly greater at T0 than at T1 (Table 4 and Table 5).

Comparison of the magnitude of root length changes (T0–T1) among Angle Class I, II, and III groups demonstrated no statistically significant differences for either endodontically treated teeth (*p* = 0.771) or vital teeth (*p* = 0.876) (Table 6).

The similarity of change scores across malocclusion classes indicates that sagittal malocclusion pattern did not appear to influence the degree of root length reduction in this cohort.

## 4. Discussion

The present single-center retrospective archival study aimed to evaluate and compare external apical root resorption in vital and endodontically treated teeth of individuals with Angle Class I, II, and III malocclusions following fixed orthodontic treatment, using digital panoramic radiography. The null hypothesis was that endodontically treated teeth respond to orthodontic forces in a manner similar to vital teeth and therefore do not pose an additional risk in terms of apical root resorption during orthodontic treatment.

The results of this study demonstrated that apical root resorption occurred in both endodontically treated teeth and their contralateral vital teeth following fixed orthodontic treatment. However, no statistically significant difference was found between the two groups with respect to the amount of resorption. These findings indicate that teeth with adequately performed root canal treatment undergo a similar degree of apical root resorption as vital teeth, thereby supporting the initial hypothesis. Furthermore, the standardized mean difference between groups was negligible (Cohen’s d = 0.06), indicating that the minimal observed difference between endodontically treated and vital teeth was not clinically meaningful. Although both groups demonstrated an average root length reduction of approximately 1 mm, this magnitude remains substantially below the commonly accepted threshold for clinically significant root resorption (approximately one-third of the original root length). Therefore, the observed changes are unlikely to compromise long-term tooth prognosis in properly treated cases.

For the assessment of apical root resorption following orthodontic treatment, digital panoramic radiography, intraoral periapical radiography, and (CBCT) are commonly used imaging modalities [19,21]. In the present study, digital panoramic radiographs—routinely used in orthodontic clinics—were preferred due to their advantages, including visualization of the entire dental arch and lower radiation exposure compared with CBCT [22]. To minimize potential measurement errors related to magnification and superimposition, the panoramic images were digitally calibrated, and standardization was achieved by measuring reproducible anatomical landmarks at both T0 and T1 time points. Nevertheless, despite the use of identical equipment, standardized positioning, and intra-individual comparisons, residual projection errors and geometric distortion inherent to panoramic imaging cannot be completely eliminated.

In addition, orthodontic treatment may induce changes in tooth angulation, including tipping or torque movements, which could influence the projection of the tooth’s long axis on two-dimensional panoramic images. Although measurements were performed along the radiographic long axis at both time points to maintain consistency, changes in three-dimensional tooth orientation may have introduced minor projection-related variability in linear measurements. Therefore, the potential influence of angulation changes should be considered when interpreting the magnitude of root length reduction observed in this study.

The severity of apical root resorption may also be influenced by factors related to endodontic treatment, such as the quality of the root canal filling, the integrity of the apical seal, and periapical tissue health. To ensure reliable evaluation of resorption in endodontically treated teeth, only teeth with high-quality root canal fillings and without radiographic evidence of active pathology or periapical lesions were included. In addition, individuals who did not complete orthodontic treatment and teeth with compromised incisal–occlusal edge integrity during treatment were excluded to allow accurate longitudinal measurements.

All individuals included in this study completed fixed orthodontic treatment with a mean duration of 20.3 ± 2.1 months. Apical root resorption was observed in both endodontically treated and vital teeth following this treatment period. Previous studies have reported that root resorption is closely associated with treatment duration, with increasing severity observed as treatment time is prolonged [23,24,25,26]. In the present study, the relatively moderate treatment duration may explain the limited extent of resorption detected in both tooth groups.

Although treatment duration is recognized as a potential influencing factor in orthodontically induced root resorption, no significant association was observed between individual treatment duration and resorption magnitude in the present cohort. The relatively narrow range of treatment duration (20.3 ± 2.1 months) may have limited the variability necessary to detect a duration-dependent effect.

In addition to endodontic treatment quality and orthodontic treatment duration, apical root resorption is a multifactorial process influenced by biological variables such as genetic predisposition, root morphology, tooth type, and patient age, as well as mechanical factors including the type and magnitude of the applied orthodontic forces. Tooth type and jaw location may therefore act as potential confounding factors, given their distinct anatomical characteristics and biomechanical responses, and should be considered when interpreting the findings.This multifactorial nature likely contributes to the ongoing controversy in the literature regarding the response of endodontically treated teeth to orthodontic forces.

A limited number of studies have reported greater root resorption in endodontically treated teeth. Gong et al. suggested that root canal treatment may weaken the defense mechanisms of cementum tissue, thereby increasing susceptibility to resorptive activity [16], while Wickwire et al. similarly reported greater resorption in endodontically treated teeth [15].

Conversely, the absence of pulp tissue in endodontically treated teeth may limit the production of inflammatory mediators, which could reduce osteoclastic activity and lead to less root resorption. Kurnaz and Büyükçavuş reported that following orthodontic treatment, root length in vital teeth was significantly shorter compared with endodontically treated teeth [23], and Khan et al. also found that average root resorption was greater in vital teeth [27]. These findings were further supported by Kolcuoğlu et al. and Lee et al. [28,29].

In line with the findings of the present study, Mattison et al. reported no significant difference in root resorption between vital and endodontically treated teeth and concluded that the presence or absence of pulp tissue does not significantly influence the resorptive process [30]. Irvin and Eliezer reported mean resorption values of 0.84 mm in endodontically treated teeth and 0.78 mm in vital teeth, stating that this difference was neither statistically nor clinically significant [31]. Castro et al. supported these findings using CBCT-based evaluations [32]. These conflicting findings further highlight the multifactorial nature of apical root resorption and the influence of both biological and mechanical factors.

The severity of root resorption has also been associated with the type and duration of orthodontic forces as well as the mechanics applied, which may vary according to malocclusion class. While relatively light orthodontic forces are often sufficient in Angle Class I cases, Angle Class II and III malocclusions may require more complex mechanics, intermaxillary elastics, or longer treatment durations due to underlying skeletal discrepancies. In the present study, however, no significant differences were found in the amount of apical root resorption between Angle Class I, II, and III groups in either endodontically treated or vital teeth. Although previous studies have suggested that Class II malocclusions or the use of intermaxillary elastics may increase the risk of resorption [33,34,35,36], the number of studies comparing root resorption among different malocclusion classes remains limited.

### 4.1. Limitations of the Study

Several limitations of this study should be acknowledged. Although each patient contributed only one contralateral tooth pair, thereby preserving statistical independence, the paired design inherently limits generalizability to other teeth within the same individual.Potential selection bias inherent to retrospective archival studies should be considered, as the sample was restricted to patients with complete radiographic records fulfilling the predefined inclusion criteria. The retrospective design and relatively limited sample size may restrict the generalizability of the findings, and the observational nature of the study precludes any causal inference regarding the relationship between endodontic status and the extent of apical root resorption. In addition, detailed archival records regarding the magnitude, direction, and duration of the orthodontic forces and mechanics applied were not available. Since T1 measurements represent post-treatment outcomes, the observed apical root resorption reflects the cumulative biological response to the orthodontic mechanics applied; however, variations in force systems may have influenced the degree of resorption.

Furthermore, panoramic radiographs provide two-dimensional data and are subject to inherent magnification and distortion, which should be considered when interpreting the results. Additionally, although intra-individual contralateral comparisons were performed, different tooth types (molars, premolars, canines, and incisors) were included in the measurements. Given their distinct anatomical characteristics and biomechanical responses, this heterogeneity should be considered when interpreting the findings.

Subgroup analyses were performed according to tooth type; however, the study was not specifically powered for stratified comparisons among incisors, canines, premolars, and molars. Pooling different tooth categories in the primary analysis may introduce biomechanical heterogeneity, as root morphology, root surface area, and force distribution patterns vary among tooth types. Therefore, the results should be interpreted with caution when extrapolated to individual tooth categories. Larger studies specifically designed and powered for tooth-type stratification are warranted to provide more definitive conclusions.

Similarly, additional subgroup analyses were conducted according to jaw location; however, the study was not specifically powered to detect small differences between maxillary and mandibular teeth. Therefore, these findings should also be interpreted with caution.

### 4.2. Recommendations for Future Research

Nevertheless, as the study was conducted in a single center, standardized diagnostic, treatment, and follow-up protocols were applied, which can be considered a methodological advantage in terms of internal consistency. The standardization of patient records, imaging equipment, and measurement methods contributes to methodological reliability.

However, the single-center retrospective design may limit the generalizability of the findings to other clinical environments with different operator techniques, treatment protocols, or patient characteristics.

Therefore, future well-designed prospective studies incorporating three-dimensional imaging modalities and detailed documentation of orthodontic force systems are required to better elucidate the relationship between malocclusion type, orthodontic mechanics, and apical root resorption. Larger multicenter studies with stratification according to tooth type and arch location would further clarify potential confounding factors and improve the external validity of the findings.

## 5. Conclusions

Endodontically treated teeth and vital teeth demonstrated comparable magnitudes of external apical root resorption following fixed orthodontic treatment, with no statistically significant difference detected between groups. However, given the retrospective design and the use of two-dimensional panoramic radiography, the findings should be interpreted with caution. Within these methodological limitations and the statistical power of the present study, properly treated endodontic teeth were not associated with a clinically meaningful increase in apical root resorption during orthodontic therapy.

From a clinical perspective, these findings suggest that endodontically treated teeth do not appear to present an increased risk of clinically relevant apical root resorption under the conditions evaluated in this study. Nevertheless, individualized treatment planning and careful radiographic monitoring remain essential, and prospective studies using three-dimensional imaging modalities are warranted to further confirm these observations.

## Figures and Tables

**Table 1:** Distribution of tooth types and jaw location in the study sample.

Tooth Type	Jaw	ETT (n)	VT (n)	Total (n)
Molars	Maxilla	11	11	22
	Mandible	12	12	24
Premolars	Maxilla	9	9	18
	Mandible	8	8	16
Canines	Maxilla	5	5	10
	Mandible	6	6	12
Incisors	Maxilla	4	4	8
	Mandible	5	5	10
Total		60	60	120

n = number of teeth. Each of the 60 patients contributed one endodontically treated tooth and one contralateral vital tooth for intra-individual comparison.

**Table 2:** Comparison of pre-treatment (T0) and post-treatment (T1) root length measurements of endodontically treated and vital teeth.

Measurements	T0	T1	Difference (T0–T1)	*p*
	Mean ± SD	Mean ± SD		
ETT (mm)	41.25 ± 6.28	40.23 ± 6.04	1.02 ± 1.36	<0.001
VT (mm)	40.38 ± 5.98	39.27 ± 5.91	1.11 ± 1.79	<0.001

Data are presented as mean ± standard deviation (SD). ETT: Endodontically treated teeth; VT: Vital teeth. Intra-group comparisons were performed using the Wilcoxon signed-rank test.

**Table 3:** Comparison of root length reduction (T0–T1) between groups.

Difference (T0–T1)	ETT (n = 60)	VT (n = 60)	*p*	Cohen’s d
Mean ± SD (mm)	1.02 ± 1.36	1.11 ± 1.79	0.718	0.06
Median (IQR) (mm)	0.75 (0.18–1.45)	0.50 (0.10–1.48)		
95% CI of mean	0.68–1.37	0.66–1.56		

Data are presented as mean ± standard deviation (SD) and median (interquartile range, IQR). ETT: Endodontically treated teeth; VT: Vital teeth. Intra-group comparisons were performed using the Wilcoxon signed-rank test. Between-group comparisons of change scores (ETT vs. VT) were performed using the Mann–Whitney U test. Cohen’s d represents the standardized mean difference between groups.

**Table 4:** Comparison of pre-treatment (T0) and post-treatment (T1) root length measurements of endodontically treated teeth according to angle malocclusion classification.

ETT (mm)	Class I (n = 20)	Class II (n = 20)	Class III (n = 20)	*p*
	Mean ± SD	Mean ± SD	Mean ± SD	
T0	39.83 ± 5.94	43.48 ± 6.10	40.43 ± 6.48	0.108
T1	38.82 ± 5.23	42.46 ± 6.02	39.41 ± 6.45	0.093

Data are presented as mean ± standard deviation (SD). ETT: Endodontically treated teeth; n: Number of subjects in each Angle malocclusion group. Intergroup comparisons were performed using the Kruskal–Wallis test. (*p* values in the last column). Within-group comparisons (T0 vs T1) were performed using the Wilcoxon signed-rank test: Class I: *p* = 0.011; Class II: *p* = 0.002; Class III: *p* < 0.001.

**Table 5:** Comparison of pre-treatment (T0) and post-treatment (T1) root length measurements of vital teeth according to angle malocclusion classification.

VT (mm)	Class I (n = 20)	Class II (n = 20)	Class III (n = 20)	*p*
	Mean ± SD	Mean ± SD	Mean ± SD	
T0	39.01 ± 3.99	42.44 ± 6.03	39.69 ± 7.20	0.163
T1	37.95 ± 3.75	41.49 ± 5.99	38.38 ± 7.13	0.080

Data are presented as mean ± standard deviation (SD). VT: Vital teeth; n: Number of subjects in each Angle malocclusion group. Intergroup comparisons were performed using the Kruskal–Wallis test. (*p* values in the last column). Within-group comparisons (T0 vs T1) were performed using the Wilcoxon signed-rank test: Class I: *p* = 0.005; Class II: *p* = 0.022; Class III: *p* = 0.001.

**Table 6:** Comparison of T0–T1 difference measurements in endodontically treated and vital teeth between angle class I, II and III Groups.

Difference (T0–T1)	Class I (n = 20)	Class II (n = 20)	Class III (n = 20)	*p*
	Mean ± SD	Mean ± SD	Mean ± SD	
ETT (mm)	1.01 ± 1.83	1.02 ± 1.33	1.02 ± 0.77	0.771
VT (mm)	1.06 ± 1.58	0.96 ± 1.64	1.31 ± 2.17	0.876

Data are presented as mean ± standard deviation (SD). ETT: Endodontically treated teeth; VT: Vital teeth; n: Number of subjects in each Angle malocclusion group. Intergroup comparisons were performed using the Kruskal–Wallis test.

## Data Availability

The data presented in this study are not publicly available due to ethical and privacy restrictions, as they are derived from anonymized patient radiographic records obtained from institutional archives. Data may be made available from the corresponding author upon reasonable request and with permission of the relevant ethics committee.

## Supplementary Materials

The following supporting information can be downloaded at: https://www.mdpi.com/article/10.3390/jcm15051963/s1, File S1: STROBE Statement—Checklist of items that should be included in reports of cohort studies.

## Abbreviations

CBCT	Cone-Beam Computed Tomography
CEJ	Cementoenamel Junction
EARR	External Apical Root Resorption
ETT	Endodontically Treated Teeth
VT	Vital Teeth

## References

[1] Bakland L.K. (1992). Root resorption. Dent. Clin. N. Am..

[2] Brezniak N., Wasserstein A. (2002). Orthodontically induced inflammatory root resorption. Part I: The basic science aspects. Angle Orthod..

[3] Krishnan V., Davidovitch Z. (2006). Cellular, molecular, and tissue-level reactions to orthodontic force. Am. J. Orthod. Dentofac. Orthop..

[4] Brudvik P., Rygh P. (1993). Non-clast cells start orthodontic root resorption in the periphery of hyalinized zones. Eur. J. Orthod..

[5] Brudvik P., Rygh P. (1993). The initial phase of orthodontic root resorption incident to local compression of the periodontal ligament. Eur. J. Orthod..

[6] Harris E.F., Kineret S.E., Tolley E.A. (1997). A heritable component for external apical root resorption in patients treated orthodontically. Am. J. Orthod. Dentofac. Orthop..

[7] Killiany D.M. (1999). Root resorption caused by orthodontic treatment: An evidence-based review of literature. Semin. Orthod..

[8] Brezniak N., Wasserstein A. (2016). Orthodontitis: The inflammation behind tooth movement and orthodontic root resorption. Biology of Orthodontic Tooth Movement.

[9] Harry M.R., Sims M.R. (1982). Root resorption in bicuspid intrusion. A scanning electron microscope study. Angle Orthod..

[10] Stenvik A., Mjör I.A. (1970). Pulp and dentine reactions to experimental tooth intrusion. A histologic study of the initial changes. Am. J. Orthod..

[11] Reitan K. (1974). Initial tissue behavior during apical root resorption. Angle Orthod..

[12] Sameshima G.T., Sinclair P.M. (2001). Predicting and preventing root resorption: Part I. Diagnostic factors. Am. J. Orthod. Dentofac. Orthop..

[13] Krishnan V. (2005). Critical issues concerning root resorption: A contemporary review. World J. Orthod..

[14] Acharya N., Shrestha R., Maskey S., Yadav R. (2022). Endodontic considerations in contemporary orthodontic practice. Orthod. J. Nepal.

[15] Wickwire N.A., McNeil M.H., Norton L.A., Duell R.C. (1974). The effects of tooth movement upon endodontically treated teeth. Angle Orthod..

[16] Gong X.Y., Jian X.C., Lei Y.H., Yao Z.Y., Hong T., Ma D.Y. (2007). Effects of root canal treatment on orthodontic movement in cat cuspids. Shanghai Kou Qiang Yi Xue.

[17] Malmgren O., Goldson L., Hill C., Orwin A., Petrini L., Lundberg M. (1982). Root resorption after orthodontic treatment of traumatized teeth. Am. J. Orthod..

[18] Satoh I. (1990). Root resorption of vital and endodontically treated teeth in orthodontic movement. J. Kanagawa Odontol. Soc..

[19] Esteves T., Ramos A.L., Pereira C.M., Hidalgo M.M. (2007). Orthodontic root resorption of endodontically treated teeth. J. Endod..

[20] Spurrier S.W., Hall S.H., Joondeph D.R., Shapiro P.A., Riedel R.A. (1990). A comparison of apical root resorption during orthodontic treatment in endodontically treated and vital teeth. Am. J. Orthod. Dentofac. Orthop..

[21] Qin F., Zhou Y. (2019). The influence of bracket type on the external apical root resorption in class I extraction patients: A retrospective study. BMC Oral Health.

[22] Marinescu I.R., Bănică A.C., Mercuţ V., Gheorghe A.G., Drăghici E.C., Cojocaru M.O., Scrieciu M., Popescu S.M. (2019). Root resorption diagnostic: Role of digital panoramic radiography. Curr. Health Sci. J..

[23] Kurnaz S., Buyukcavus M.H. (2021). External root resorption in root-filled and vital teeth after extraction and non-extraction orthodontic treatments: A split-mouth retrospective study. Acta Odontol. Scand..

[24] Segal G.R., Schiffman P.H., Tuncay O.C. (2004). Meta analysis of the treatment-related factors of external apical root resorption. Orthod. Craniofac. Res..

[25] Fox N. (2005). Longer orthodontic treatment may result in greater external apical root resorption. Evid. Based Dent..

[26] Liu Z., Ouyang Y., Lou Y., Han Y., Lu M., Yu M., Wang H., Ding W. (2025). Orthodontically induced root resorption in endodontically treated and vital teeth: A cone beam computed tomographic study. Prog. Orthod..

[27] Khan A.R., Fida M., Shaikh A. (2018). Apical root resorption in endodontically treated teeth. J. Ayub Med. Coll. Abbottabad.

[28] Kolcuoğlu K., Oz A.Z. (2020). Comparison of orthodontic root resorption of root-filled and vital teeth using micro-computed tomography. Angle Orthod..

[29] Lee Y.J., Lee T.Y. (2016). External root resorption during orthodontic treatment in root-filled teeth and contralateral teeth with vital pulp: A clinical study of contributing factors. Am. J. Orthod. Dentofac. Orthop..

[30] Mattison G.D., Delivanis H.P., Delivanis P.D., Johns P.I. (1984). Orthodontic root resorption of vital and endodontically treated teeth. J. Endod..

[31] Irvin H.G., Eliezer G.L. (2019). Comparison of external root resorption after orthodontic treatment. Int. J. Fam. Community Med..

[32] Castro I., Valladares-Neto J., Estrela C. (2015). Contribution of cone beam computed tomography to the detection of apical root resorption after orthodontic treatment in root-filled and vital teeth. Angle Orthod..

[33] McFadden W.M., Engstrom C., Engstrom H., Anholm J.M. (1989). A study of the relationship between incisor intrusion and root shortening. Am. J. Orthod. Dentofac. Orthop..

[34] Linge L., Linge B.O. (1991). Patient characteristics and treatment variables associated with apical root resorption during orthodontic treatment. Am. J. Orthod. Dentofac. Orthop..

[35] Levander E., Malmgren O., Stenback K. (1998). Apical root resorption during orthodontic treatment of patients with multiple aplasia: A study of maxillary incisors. Eur. J. Orthod..

[36] Brin I., Tulloch J.F., Koroluk L., Philips C. (2003). External apical root resorption in Class II malocclusion: A retrospective review of 1-versus 2-phase treatment. Am. J. Orthod. Dentofac. Orthop..

